# Predicting Human Papillomavirus Vaccination Uptake in Saudi Arabia: Analyzing Health Belief Model Constructs, Vaccine Hesitancy, and Pap Smear Uptake

**DOI:** 10.3390/vaccines14060521

**Published:** 2026-06-10

**Authors:** Faten A. AlRadini, Joud Mohammed Alibrahim, Roqaya Saud Almasoud, Sarah Abdullah Alsubaie, Arub Magid Althbety, Ghofran Hadi Alqahtani, Rahil Esmail Alshanqiti, Layan Mohammed Kashm, Danah Abdullah Aljahdali, Amel Fayed

**Affiliations:** Department of Family and Community Medicine, College of Medicine, Princess Nourah bint Abdulrahman University, P.O. Box 84428, Riyadh 11671, Saudi Arabia; faalradini@pnu.edu.sa (F.A.A.); 443009088@pnu.edu.sa (J.M.A.); 443000622@pnu.edu.sa (R.S.A.); 443000835@pnu.edu.sa (S.A.A.); 443000909@pnu.edu.sa (A.M.A.); 442003502@pnu.edu.sa (G.H.A.); 442000977@pnu.edu.sa (R.E.A.); 442002005@pnu.edu.sa (L.M.K.); 443000905@pnu.edu.sa (D.A.A.)

**Keywords:** Health Belief Model, Saudi Arabia, HPV vaccine, cervical cancer, pap smear

## Abstract

**Background**: Cervical cancer is among the most common cancers affecting women worldwide, with high morbidity and mortality in low- and middle-income countries. In Saudi Arabia, most cases are diagnosed at a late stage despite the availability of free HPV vaccination and screening. **Objectives**: To identify Saudi women’s perceptions of the HPV vaccine using the Health Belief Model, estimate willingness to receive the HPV vaccine and the factors influencing it, assess uptake of Pap smear and HPV vaccine, and define barriers to both practices. **Methodology**: A cross-sectional study of a convenience sample of 1334 Saudi women aged 16 to 65 years, from all regions of Saudi Arabia, was conducted. Data were collected via an online questionnaire that included sociodemographic characteristics, beliefs about the HPV vaccine based on the Health Belief Model, vaccine hesitancy, and HPV vaccine and Pap smear uptake. Data were analyzed using SPSS version 29. **Results**: Only 6% completed their vaccination series or received at least one dose; 37.3% planned to get vaccinated; and 56.7% stated they do not intend to get vaccinated. The main reasons for vaccine refusal were lack of trust (41.8%) and fear of side effects (32.3%). Only 21% had undergone Pap smear testing, with barriers including embarrassment and fear. Among the HBM constructs, perceived susceptibility, benefits, and barriers remained statistically significant predictors of HPV vaccination. Increased perceived susceptibility and benefits raise the likelihood of accepting the HPV vaccine, while higher perceived barriers lessen it. Vaccine hesitancy had a significant negative effect on willingness to receive the HPV vaccine (OR = 0.78, 95% CI 0.69–0.90, *p* < 0.01). Additionally, Pap smear uptake was an independent predictor of the intent to get the HPV vaccine (OR = 1.78, 95% CI 1.25–2.54, *p* < 0.01). The independent factors influencing HPV vaccine uptake were largely similar to those affecting the willingness to receive the vaccine, except for age, perceived benefits, and Pap smear uptake. **Conclusions**: There is a gap between Saudi women’s intention to get HPV vaccinated and actual vaccination. Women who saw a high risk of HPV-related cancer, believed in vaccine efficacy, had a Pap smear, and were open to vaccination were more likely to vaccinate. Hesitant women and those perceiving barriers were less likely to vaccinate or consider it. The main gaps for future campaigns are perceptions of HPV severity and cultural factors influencing decision-making. Emphasizing HPV as a cancer-related virus rather than a sexually transmitted infection can reduce barriers and highlight its severity.

## 1. Background

Cervical cancer (CC) is defined as the presence of malignant cells in any layer of the cervix. It is the fourth leading cause of cancer-related death in women worldwide [1], with around 660,000 new cases and nearly 350,000 deaths in 2022 [2]. Almost all cases of CC are attributable to the human papillomavirus (HPV), a sexually transmitted pathogen. After HPV infection, progression to CC occurs after a latent period of 10 to 20 years. Fifteen distinct HPV strains can cause cervical cancer, with HPV 16 and 18 accounting for approximately 70% of cases. Consequently, immunization against HPV types 16 and 18 markedly reduces the risk of developing cervical cancer, and HPV vaccination is considered as the most cost-effective public health strategy for its prevention [3,4,5].

The CC screening facilitates the early detection of cervical cancer. Pap smear screening, which detects cytological abnormalities in the cervical transformation zone, has contributed to a 70% reduction in both the incidence and mortality rates of CC in developed nations within three years of the implementation of screening programs [6].

The World Health Organization (WHO) Global Strategy defines CC elimination as reducing new cases to four or fewer per 100,000 women annually. It sets three targets to be achieved by 2030 to guide all nations toward elimination in the coming decades: 90% of girls vaccinated with the HPV vaccine by age 15; 70% of women screened using a high-performance test at ages 35 and 45; and 90% of women diagnosed with cervical precancer or cancer receiving appropriate treatment [2].

In Saudi Arabia, CC ranks as the eighth most prevalent malignancy among women aged 15 to 44 years. Approximately 10.3 million women aged 15 years and above in the country are considered at risk of developing CC [7]. Recent data indicate a concerning rise in incidence in Saudi Arabia, with approximately 358 new cases and 179 deaths annually [8]. Approximately 70% of women with CC in Saudi Arabia are diagnosed at advanced stages and have not previously undergone HPV screening [9,10].

In 2023, the Saudi Public Health Authority (SPHA) issued guidelines that include HPV vaccination and CC screening. They recommend screening for women 21–65 starting after marriage, with cytology (Pap smear) every three years and co-testing (cytology and HPV) every five years for women 30–65 who prefer extended intervals. The Saudi Food and Drug Administration (SFDA) approved the HPV vaccine in 2010 for females 11–26, but it was not included in the routine schedule until 2019. In 2022, the Ministry of Health (MOH) launched a school-based HPV vaccination program. The 2023 SPHA guidelines also advise a two-dose HPV vaccination for girls 6–17, aligned with the national schedule for ages 11–12, and a three-dose catch-up for women 15–26 at 0, 1–2, and 6 months [11,12].

Despite these concerns and initiatives, recent research indicates low uptake of screening and vaccination. In 2024, a study of 8194 Saudi women found that only 22.5% had undergone a Pap smear [13]. Another review published the same year reported that only 4% of the population had received the HPV vaccine [11].

This late-stage diagnosis and limited uptake of both screening and vaccination raise critical questions about the efficacy of current initiatives and the influence of individual perceptions of screening and protective measures.

The aim of this study is to investigate the willingness and uptake of the HPV vaccine and to explore Saudi women’s perceptions, beliefs, and concerns regarding HPV vaccination, using the Health Belief Model (HBM) to better understand the factors underlying low vaccination rates among women, who are the primary recipients of these healthcare initiatives. The study also examines the influence of vaccine hesitancy on the decision to receive the HPV vaccine and the relationship between Pap smear uptake and HPV vaccination among young Saudi women.

## 2. Methods

### 2.1. Study Design, Participants, and Setting

This study used a cross-sectional design with an online survey of Saudi women aged 16 to 65 years conducted from January to March 2025. We included women aged 16 years and older to assess both HPV vaccine uptake and willingness to receive the vaccine among Saudi women. This age group includes young women who fall within Saudi Arabia’s catch-up vaccination recommendations (15–26 years). At the same time, including older women allowed us to explore HPV-related awareness, attitudes, and preventive practices, including Pap smear screening, across a broader age range.

### 2.2. Sampling Technique and Sample Size

A convenience sampling approach was used to recruit Saudi women aged 16 to 65 years. The self-administered questionnaire was distributed across multiple platforms to maximize reach and enhance representativeness across regions.

The required sample size was calculated to be 1066 using G*Power version 3.1, based on a power of 0.95. The expected willingness rate for the HPV vaccine was assumed to be 50% [14], with a margin of error of ±5%. Ultimately, a total of 1334 participants were included in the study.

### 2.3. Data Collection Tool

Data were collected using a self-administered online questionnaire created in Research Electronic Data Capture (REDCap) [15]. REDCap is a secure, web-based software platform designed to support data capture for research studies. The questionnaire was distributed on social media platforms, primarily via WhatsApp V2.25.2, X v9.67, and Telegram v11.5, to ensure broad accessibility. The questionnaire consisted of the following sections:

#### 2.3.1. Sociodemographic Data

This section collected essential demographic information, including age, place of residence, marital status, education level, employment status, and income.

#### 2.3.2. HPV Vaccine HBM [16]

The HBM scale used in this section was adapted from a validated 12-item scale. Content and face validity were assessed by a panel of experts. This section assessed participants’ beliefs about HPV vaccination using the HBM framework. It evaluated key constructs, including perceived susceptibility, severity, barriers, benefits, and motivation. Reliability for each construct was measured using Cronbach’s alpha: perceived susceptibility (α = 0.9), perceived severity (α = 0.8), perceived benefits (α = 0.9), perceived barriers (α = 0.7), and motivation (α = 0.8).

Participants’ responses were scored using a 5-point Likert scale ranging from 1 (strongly disagree) to 5 (strongly agree). Scores for each HBM construct were computed by summing the responses for the items within that construct. For analysis, the median was used to categorize each participant as high or low for each construct.

#### 2.3.3. Vaccine Hesitancy [17]

Participants’ self-reported vaccine hesitancy was assessed using the (WHO) definition and three questions: “Have you ever refused a vaccine for yourself or a child because you considered it to be useless or dangerous?”, “Have you ever postponed a vaccine recommended by a physician due to doubts about it?” and “Have you ever accepted a vaccine for a child or yourself despite doubts regarding its efficacy?” Participants who answered yes to any of these questions were classified as “vaccine-hesitant.”

#### 2.3.4. Assessing the Uptake of the HPV Vaccine and Pap Smear

This section began with a direct question about whether participants had ever had a Pap smear. Follow-up questions explored reasons individuals may have chosen not to undergo the test, including perceived pain and cost. Similarly, the HPV vaccine section asked about vaccination status, reasons for non-participation, and factors influencing an individual’s decision to receive the vaccine.

#### 2.3.5. Willingness and Uptake of HPV Vaccination

We assessed willingness to receive the vaccine with a single direct question about vaccination status. Answer options were: “I received all my vaccination shots,” “I received at least one dose,” “I am planning to receive it soon,” or “I am not planning to get the vaccine.” Participants who reported that they are not planning to receive the vaccine were considered “unwilling to get vaccinated.” Participants who reported receiving one dose or the full series of the HPV vaccine were considered vaccine recipients.

The questionnaire was prepared in English. After assessing content and face validity, it was translated into Arabic and then back-translated into English. Further wording refinements were made after piloting the questionnaire to ensure its suitability and ease of comprehension for the Saudi population.

### 2.4. Statistical Analysis

Descriptive statistics, including means, standard deviations, medians, and interquartile ranges, were used to summarize sample characteristics. Quantitative data were analyzed using a t-test, and associations among qualitative variables were assessed using a chi-square test. A logistic regression model was used to identify independent factors associated with willingness to receive the HPV vaccine and actual vaccine uptake. Factors that showed statistically significant associations in the bivariate analysis were considered in the regression models. Adjusted odds ratios (AORs) with their corresponding 95% confidence intervals (95% CIs) were reported. A *p*-value less than 0.05 was considered statistically significant. All statistical analyses were conducted using SPSS version 29.

## 3. Results

Table 1 lists the characteristics of the study population. The study included 1334 participants. The mean age was 30.98 years (SD = 12.79). Participants were categorized into three age groups, with the majority (42.8%) aged 20–30 years. The highest level of attained education varied, with more than half holding a university degree. Most respondents resided in the central region (65.7%). Regarding income, the highest proportion (56.6%) reported having “enough” income. Among employment categories, the largest group (43.3%) was students.

Vaccine hesitancy and HPV vaccine uptake patterns are displayed in Table 2. Overall, the vaccine hesitancy rate among participants was 55.2%. Regarding HPV vaccination, only 6% completed their vaccination series or received at least one dose; 37.3% planned to get vaccinated; and 56.7% stated that they do not intend to get vaccinated.

**Figure 1:**
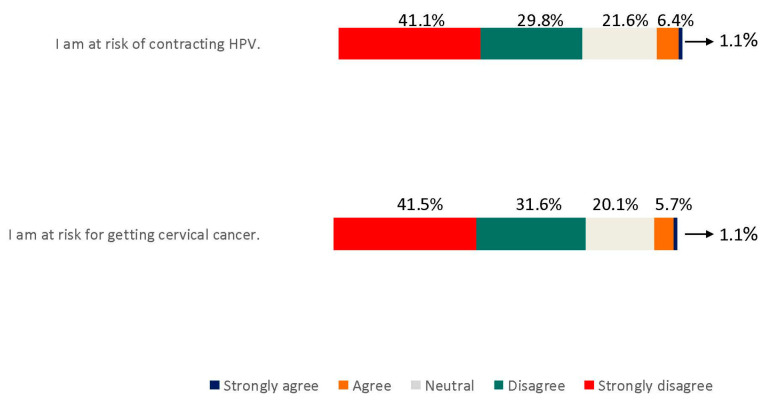
Participants’ perceived susceptibility to HPV and cervical cancer.

**Figure 2:**
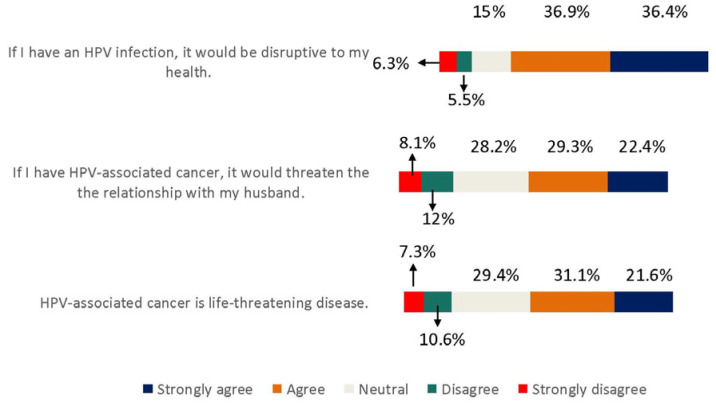
Participants’ perceived severity of HPV and cervical cancer.

**Figure 3:**
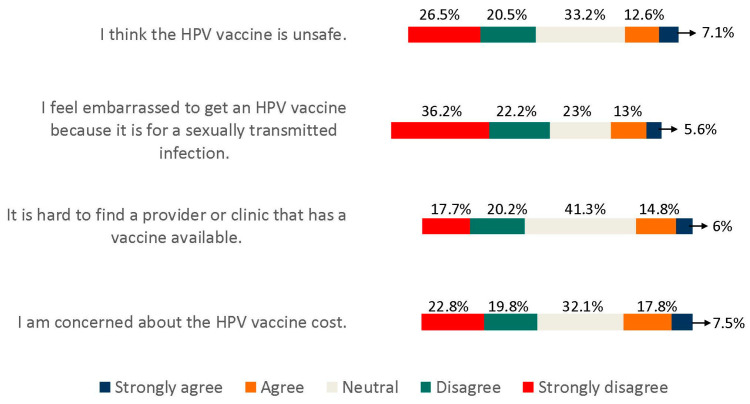
Participants’ perceived barriers to the HPV vaccine.

**Figure 4:**
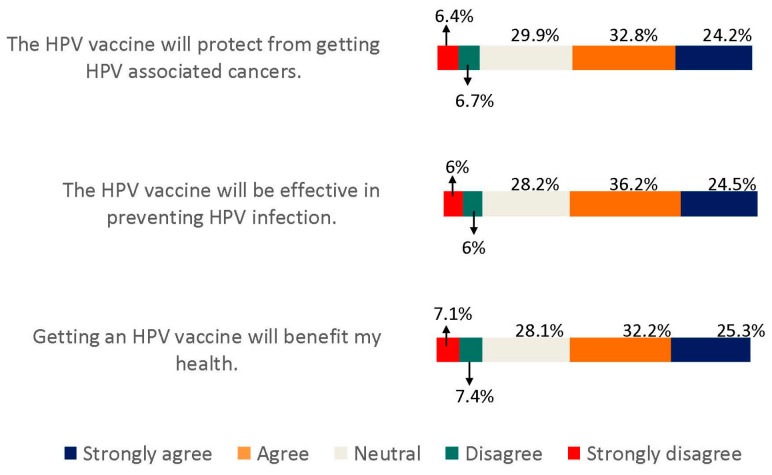
Participants’ perceived benefits of the HPV vaccine.

**Figure 5:**
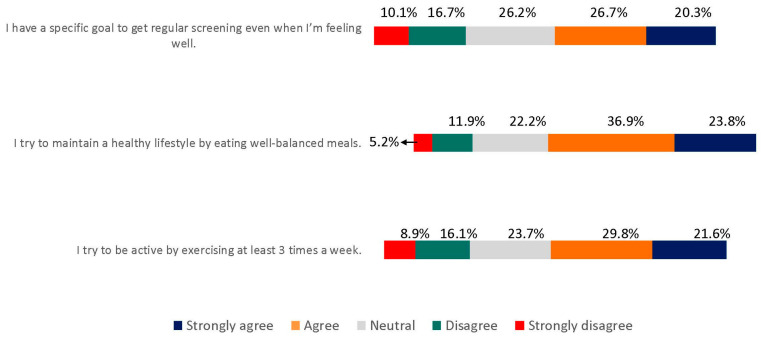
Participants’ health motivations.

Figure 1, Figure 2, Figure 3, Figure 4 and Figure 5 present the five HBM constructs assessed in this study. Figure 1 shows that only about 7% of respondents felt highly susceptible to contracting HPV or developing cervical cancer. Figure 2 presents perceived severity, with a notable share of participants recognizing HPV infection and its related cancers as serious health concerns; for instance, 73% agreed that an HPV infection would significantly disrupt their health. Figure 3 indicates that fewer than 20% of participants believed the HPV vaccine was unsafe or difficult to access, and that embarrassment and cost were not prominent concerns. Figure 4 illustrates the perceived benefits of the HPV vaccine, with more than half of participants agreeing that it protects against HPV-related cancers, prevents infection, and contributes to overall health. Finally, Figure 5 highlights participants’ health motivation, which appeared moderate overall, with over 40% reporting efforts to eat balanced meals.

Table 3 shows the association between willingness to get the HPV vaccine and various factors. Among young participants, although they were the most likely to receive the vaccine (8.4%), they also had the highest level of unwillingness to receive it (63.7%). Unmarried participants received it more often (7.4%) than married individuals (4.2%). Participants with a family history of cervical cancer were more likely to receive the HPV vaccine (12.96%) and more willing to get it (44.4%) compared to those without such a history. Additionally, nearly two-thirds of those who did not undergo a Pap smear are unwilling to receive the HPV vaccine, whereas more than half of those who had a Pap smear either already received the vaccine or are willing to do so (*p* < 0.01). Vaccine hesitancy played a significant role, with vaccine-hesitant individuals being considerably less willing (60.2%) than non-hesitant participants (52.5%). Furthermore, those who believed they were highly susceptible had a 48.4% vaccination or willingness rate, vs. 41.0% for those who believed they were low-susceptible (*p* ≤ 0.01). Similarly, 48.6% of those with high perceived severity were willing or had already been vaccinated, compared to 38.2% with lower perceived severity (*p* < 0.01). Participants with high perceived barriers were less likely to be vaccinated, whereas those with high perceived benefits were more likely to be vaccinated.

Table 4 shows the uptake of Pap smear tests and HPV vaccination, along with the reasons women reported for refusing them. Most respondents (79%) had not had a Pap smear test. The most common reasons for not taking the test were embarrassment (53.1%) and fear of pain (47.4%), followed by a lack of community information (39.1%) and the belief that they are healthy (38.6%). Fear of screening results and past negative experiences also played a role, while structural barriers like not having a female screener (23.5%) or a provider recommendation (19.6%) were less common. Regarding HPV vaccination, the main reasons for refusal included lack of trust (41.8%), fear of side effects (32.3%), and lack of knowledge (26.3%). The primary motivators for choosing to vaccinate were influence from family or friends (51%), personal decision (49.7%), and a healthcare provider’s recommendation (48.5%). Public education and personal or family history of HPV-related conditions also influenced decisions.

Results from the logistic regression analysis of factors influencing vaccine uptake and willingness to receive the HPV vaccine are shown in Table 5. After adjusting for all variables, the effects of age, perceived severity, and motivations were no longer significant. Among the HBM constructs, perceived susceptibility, benefits, and barriers remained statistically significant predictors of HPV vaccination. Increased perceived susceptibility and benefits raise the likelihood of accepting the HPV vaccine, while higher perceived barriers lessen it. Vaccine hesitancy had a significant negative effect on willingness to receive the HPV vaccine (OR = 0.78, 95% CI 0.69–0.90, *p* < 0.01). Additionally, Pap smear uptake was an independent predictor of the intent to get the HPV vaccine (OR = 1.78, 95% CI 1.25–2.54, *p* < 0.01).

The independent factors influencing HPV vaccine uptake were largely similar to those affecting the willingness to receive the vaccine, except for age, perceived benefits, and Pap smear uptake. Younger individuals were less likely to get vaccinated (OR = 0.94, 95% CI 0.91–0.97, *p* = 0.01). Perceived benefits from vaccination and prior experience with Pap smears were not significantly associated with HPV vaccine uptake.

## 4. Discussion

Our study examined HPV uptake and intentions to get vaccinated, along with Saudi women’s perceptions of the HPV vaccine, using the HBM. The findings showed low HPV vaccine uptake, and many expressed no intention to receive it. While some were open to vaccination, especially those with high perceived susceptibility to CC, those who believed the HPV vaccine is beneficial, and those who had undergone Pap smear screening for CC, others remained hesitant or unwilling to be vaccinated, particularly those generally hesitant to receive vaccines and those who perceived many barriers to vaccination.

The gap between intention and observed behavior is a well-known dilemma in health behavior research. The HPV vaccine uptake in the current study was approximately 6%, while almost one-third expressed willingness to receive it soon. Population-based studies conducted in Saudi Arabia have indicated low HPV vaccine uptake, ranging from 3% to 7.6% [18,19], with one study reporting a comparatively higher figure of 20%, but among healthcare workers [20].

Saudi Arabia has already established the program for HPV vaccination and the vaccine is considered accessible for both citizens and expats; however, the uptake rates are still less than many high-income nations such as Australia and Sweden, with over 80% coverage [21,22].

In fact, in regions with high vaccine accessibility, personal factors like knowledge and willingness are likely crucial determinants of vaccination behavior. Conversely, in areas where vaccine access is limited, boosting supply is the essential first step to encourage vaccination [14].

In the current study, alarmingly, more than half of the participants did not intend to receive the vaccine. Participants reported that skepticism about vaccine efficacy, safety or fear of infection after vaccination were the most common reasons for refusing vaccination. These perceptions were reflected in perceived barriers to the HPV vaccine in the HBM, which significantly reduced the probability of vaccine uptake or intention to receive it, and were also mirrored in the perceived benefits, where participants who perceived the HPV vaccine as beneficial were more likely to take it or consider taking it soon. This gap in awareness creates opportunities for health education campaigns and activities to correct these misconceptions and raise public awareness about the safety and effectiveness of the HPV vaccine.

The study participants agreed on a set of reasons and obstacles that can hinder vaccination, all of which were related to cultural perspectives. Parental or husband refusal, stigma about linking HPV to sexual activity, and fear of spoiling marital relations if developing HPV-related cancers were all deeply rooted in refusal and hesitancy to take the HPV vaccine. Perceived barriers significantly decreased the likelihood of vaccination uptake and the intention to receive vaccination. It is commanded in Islam to abstain from sex until after marriage. Because HPV is transmitted through sexual contact, some parents might think that vaccinating their daughters is unnecessary or immoral, fearing it could promote early sexual activity [23]. A study in Saudi Arabia revealed that religious objections accounted for 30% of vaccine opposition [24]. Research shows a strong link between religious beliefs and willingness to accept vaccines in general, including decisions related to sexually transmitted infections like HPV [23,25]. A 2024 scoping review on religious beliefs and HPV vaccine acceptance in Islamic countries clarified some important misconceptions, explored public health responses, and advised that strategies should include engaging religious leaders and communities and aligning messages with religious values to positively influence attitudes. It added that training health professionals in religious literacy and cultural competence is crucial [26].

In the present study, this is reflected in the finding that a healthcare provider’s recommendation was among the leading reported motivators for vaccination (48.5%), while a lack of provider recommendation was cited as a barrier to Pap smear testing, yet the survey was not designed to evaluate these provider-level factors directly. Evidence from Saudi Arabia further suggests that gaps in providers’ own knowledge, attitudes, and counseling practices may constrain the recommendations women receive; a national study of physicians reported important deficits in knowledge of and opinions about HPV vaccination [19], and a national study of female healthcare professionals likewise documented suboptimal HPV vaccine uptake and notable hesitancy within this group [20]. These findings indicate that provider-related factors warrant dedicated assessment rather than being inferred from recipients’ perspectives alone.

High perceived susceptibility was an independent predictor of uptake and intention to receive the HPV vaccine, indicating that beliefs about susceptibility directly influence vaccination decisions, helping identify key factors that shape women’s vaccination choices. Many studies confirmed the positive effect of high perceived susceptibility and severity of HPV on vaccine decision [27,28]. However, in the current study, perceived severity of HPV-related cancer did not significantly affect the decision to be vaccinated against HPV. The lack of the expected effect of perceived severity in encouraging women to get the HPV vaccine might be explained by a lack of knowledge about the strong relationship between HPV and cancer. It is known that the greater the individual’s perceived risk of HPV infection is, the greater their confidence in the efficacy of the HPV vaccine and their willingness to be vaccinated are [29]. Therefore, this represents an important gap that should be addressed in further educational campaigns intended to encourage women to get the HPV vaccine.

The current study population is composed mainly of students (43%) and employees (30%). Two-thirds are younger than 30 years, mostly with university degrees, representing the vast majority of young adult women in Saudi Arabia. It was alarming to find that only 36% of them were willing to take the vaccine, which is lower than the 52.9% reported in a meta-analysis of 184,351 university students [14]. This young adult population has a unique profile and a crucial role as potential health educators in community health, being the future mothers and daughters of larger families. Research on university students’ and young adults’ intentions to vaccinate indicates that universities should proactively introduce HPV education programs and involve medical and public health experts to deliver specialized lectures. The curriculum should emphasize discussion sessions to foster an interactive environment that encourages active student engagement. This strategy aims to transform students from passive recipients of information into active participants, thereby enhancing learning outcomes [30,31]. In wealthier nations, initiatives should prioritize not just spreading information but also enhancing health literacy and risk awareness to facilitate the shift from having an intention to taking action [32].

Pap smear screening and HPV vaccination are both important behaviors to protect against CC. Both behaviors are influenced by a similar set of socio-cognitive factors; if a person views the HPV vaccine as necessary, they are statistically more likely to adhere to Pap smear guidelines [33,34,35,36]. Our findings show that the rate of Pap smear test uptake matches previous research on cervical cancer screening among Saudi women [13,37]; however, this rate remains consistently lower than international figures, while the US data is over three times higher [38]. Additionally, women who had previously undergone a Pap smear were more willing to receive the HPV vaccine.

The wide range of similarities in attitudes and behaviors toward Pap smears and HPV vaccination might stem from their nature as protective rather than curative measures and from the stigma associated with linking them to sexually transmitted diseases. These similarities make them cornerstones of protection against CC, and initiatives that promote this protection can focus on both behaviors simultaneously, considering the positive attitude and uptake as synergistic with the other. Additionally, the association between the HPV vaccine, Pap smear and sexual transmission has contributed to reluctance, particularly in conservative societies such as Saudi Arabia, and framing the vaccine as a cancer-prevention tool has been effective in improving acceptance in some countries [39].

Our study also examined the key barriers that prevent women from undergoing Pap smear testing. The most reported barriers were embarrassment, perceived pain, and fear of test results, underscoring the strong influence of psychological and emotional factors on screening avoidance. The high prevalence of embarrassment suggests that cultural and social stigmas surrounding gynecological exams may discourage routine screening. Additionally, fear of a positive diagnosis can lead to avoidance behaviors, delaying early detection and treatment. Similarly, a study in the UK found that emotional barriers posed the greatest threat to cervical cancer screening attendance [40]. Other barriers included time constraints, difficulty scheduling appointments, and a lack of female providers. Low awareness and a lack of physician encouragement also hindered screening participation, underscoring the importance of physician encouragement in shaping health-seeking behaviors.

In the current study, 55% of participants reported vaccine hesitancy. Among those hesitant, 60% were unwilling to get the HPV vaccine, compared with 52% of non-hesitant individuals. Hesitancy is influenced by various factors that differ across individuals, locations, times, and vaccine types. The WHO-SAGE defines hesitation as delaying or refusing vaccination despite vaccine availability and considers it a significant global health threat and a barrier to controlling infectious diseases [41]. Research on HPV vaccine hesitancy suggests that this reluctance may be due to a lack of awareness and understanding of HPV-related illnesses, as well as limited information about the vaccine’s benefits and long-term safety. The perceived connection between HPV and sexuality may also contribute to hesitancy. Additionally, concerns about vaccine safety and misconceptions about social norms have led to lower demand. The problem extends beyond an individual’s concern, as hesitancy can also influence family decisions. For example, a 2021 study reported that adolescent HPV vaccination coverage was significantly lower among parents who were hesitant than among those who were not [42]. Addressing this hesitancy requires sharing information on safety, efficacy, legislation, and regulations to promote vaccination [43,44].

## 5. Strengths and Limitations

The current study has several strengths: it investigated the effects of all HBM constructs related to HPV vaccination and simultaneously examined how vaccine hesitancy and Pap smear uptake influence the decision to receive HPV vaccination. It also recruited a large number of young Saudi women from diverse geographic regions and socioeconomic backgrounds to mitigate the expected selection bias inherent in convenience sampling. The study compared Saudi women’s intention to get vaccinated against HPV with their actual vaccine uptake. However, we are aware that some limitations in the study, including potential selection bias from the convenience sampling technique, as reflected in the sociodemographic characteristics of the sample, may limit the generalizability of the findings to the broader population of Saudi women. Additionally, due to the lack of verification of vaccine uptake from a medical registry, we relied solely on self-reported vaccination status. We acknowledge that including women across a wide age range may have influenced our estimates of HPV vaccine uptake and willingness. Because HPV vaccination in Saudi Arabia mainly targets younger females, older women may have been less likely to receive the vaccine or consider themselves eligible for it, which could have resulted in lower overall uptake and willingness estimates. On the other hand, including older women may have increased the reported rates of Pap smear screening, as these are more relevant to adult and married women. In addition to the limitations mentioned above, the online distribution of the survey through social media platforms may have underrepresented women with limited digital literacy, lower educational attainment, or lower socioeconomic status. As these groups may face greater barriers to accessing health information and preventive healthcare services, HPV awareness and knowledge may have been overestimated in our sample. Consequently, the observed rates of vaccine hesitancy and barriers to cervical cancer screening and HPV vaccination may underestimate the true magnitude of these challenges in the broader Saudi female population. Therefore, caution is warranted when generalizing these findings to all Saudi women.

## 6. Conclusions and Recommendation

There is a significant gap between the intention to get vaccinated against HPV and the actual uptake of the vaccine among Saudi women. Women who perceived high susceptibility to HPV-related cancer and infection, considered the efficacy of the HPV vaccine, had a Pap smear, and were open to vaccination in general were more likely to get vaccinated. Vaccine-hesitant women and those who perceived many barriers to getting the HPV vaccine were less likely to take the vaccine or even consider it.

The main gap that can be targeted in future educational campaigns is related to the perceived severity of HPV infection and the barriers that influence women’s vaccination decisions. Educational interventions should provide comprehensive and scientifically accurate information regarding HPV infection, transmission, cervical cancer prevention, and the benefits of HPV vaccination. While maintaining accuracy regarding transmission routes, public health messages may be more effective when HPV vaccination is also framed within a broader cancer-prevention and women’s health context. Schools and universities may serve as important settings for improving HPV-related knowledge, addressing misconceptions, reducing stigma, and promoting informed decision-making regarding cervical cancer screening and HPV vaccination.

Future research should also directly evaluate healthcare provider-related factors, including physicians’ counseling practices, recommendation patterns, and the barriers that limit their recommendations for cervical cancer screening and HPV vaccination, as well as the impact of these factors on women’s uptake of Pap smear screening and HPV vaccination.

## Figures and Tables

**Table 1:** Characteristics of the study population.

	VARIABLE	N (%)
**AGE**	<20	193 (15.8)
	20–30	523 (42.8)
	>30	507 (41.5)
**PLACE OF RESIDENCY**	Central region	846 (65.7)
	Western region	55 (4.3)
	Eastern region	160 (12.4)
	Northern region	156 (12.1)
	Southern region	71 (5.5)
**MARITAL STATUS**	Married	605 (47.1)
	Unmarried	680 (52.9)
**EDUCATIONAL LEVEL (THE HIGHEST LEVEL OF EDUCATION SUCCESSFULLY COMPLETED)**	School degree	502 (39.0)
	University degree	723 (56.2)
	Post-graduate degree	62 (4.8)
**EMPLOYMENT STATUS**	Employed	374 (29.0)
	Unemployed	357 (27.7)
	Student	558 (43.3)
**INCOME LEVEL**	Enough and save	295 (22.9)
	Enough	729 (56.6)
	Not enough	186 (14.5)
	Not enough and in debt	77 (6.0)
**DO YOU HAVE A FAMILY HISTORY OF CERVICAL CANCER?**	No	1056 (82.1)
	Yes	54 (4.2)
	I am not sure	177 (13.8)
**HAVE YOU EVER HAD A PAP SMEAR TEST?**	No	1015 (79.0)
	Yes	269 (21.0)

Data are presented as frequency and percentage.

**Table 2:** Vaccine hesitancy and HPV vaccine uptake.

	Variables	N (%)
**Vaccine Hesitancy**		
Have you ever refused a vaccine for yourself or a child because you considered it to be useless or dangerous?	No	957 (74.7)
	Yes	324 (25.3)
Have you ever postponed a vaccine recommended by a physician because of doubt about it?	No	1005 (78.7)
	Yes	272 (21.3)
Have you ever accepted a vaccine for a child or yourself despite doubt about its efficacy?	No	735 (57.4)
	Yes	546 (42.6)
Overall vaccine hesitancy	Vaccine-hesitant	703 (55.2)
	Not vaccine-hesitant	571 (44.8)
**Have you received the HPV vaccine?**	Not willing	724 (56.7)
	Willing	476 (37.3)
	Received one dose	41 (3.2)
	Received the full course	35 (2.7)
**Willingness to receive HPV**	Unwilling	724 (56.7)
	Willing	476 (37.3)
	Received one/full doses	76 (5.9)

Data are presented as frequency and percentage.

**Table 3:** HPV vaccination and willingness to receive the vaccine across different groups in the study sample.

		Received One/Full Doses (N = 76)		Willing (N = 476)		Not Willing (N = 724)		*p*-Value
		N	%	N	%	N	%	
Age category	19 and lower	16	8.42	53	27.89	121	63.68	<0.01
	20–30	41	7.85	222	42.53	259	49.62	
	≥31	16	3.17	188	37.23	301	59.60	
Educational level	School degree	38	7.69	184	37.25	272	55.06	0.21
	University degree	35	4.87	264	36.72	420	58.41	
	Post-graduate degree	3	4.92	27	44.26	31	50.82	
Employment Status	Employed	17	4.57	138	37.10	217	58.33	0.01
	Unemployed	13	3.74	123	35.34	212	60.92	
	student	46	8.29	215	38.74	294	52.97	
Income level	Enough and save	11	3.81	115	39.79	163	56.40	0.13
	Enough	48	6.64	250	34.58	425	58.78	
	Not enough	12	6.49	73	39.46	100	54.05	
	In debt	5	6.49	37	48.05	35	45.45	
Place of residency	Central region	39	4.68	316	37.89	479	57.43	0.02
	Western region	6	11.11	26	48.15	22	40.74	
	Eastern region	13	7.98	62	38.04	88	53.99	
	Northern region	9	5.84	53	34.42	92	59.74	
	Southern region	9	13.04	19	27.54	41	59.42	
Marital status	Married	25	4.20	221	37.14	349	58.66	0.04
	Unmarried	50	7.40	255	37.72	371	54.88	
Do you have a family history of cervical cancer?	No	53	5.08	386	36.97	605	57.95	0.02
	Yes	7	12.96	24	44.44	23	42.59	
	I am not sure	16	9.14	66	37.71	93	53.14	
Have you ever had a Pap smear test?	No	63	6.26	349	34.66	595	59.09	<0.01
	Yes	13	4.87	126	47.19	128	47.94	
Vaccine hesitancy	Yes	51	7.30	227	32.47	421	60.23	<0.01
	No	25	4.42	244	43.11	297	52.47	
HBM Constructs								
Perceived susceptibility	Low	52	5.94	307	35.05	517	59.02	0.04
	High	23	5.96	164	42.49	199	51.55	
Perceived severity	Low	40	6.13	209	32.06	403	61.81	<0.01
	High	34	5.58	262	43.02	313	51.40	
Perceived barriers	Low	55	8.66	285	44.88	295	46.46	<0.01
	High	19	3.11	182	29.84	409	67.05	
Perceived benefits	Low	40	6.27	155	24.29	443	69.44	<0.01
	High	34	5.59	313	51.48	261	42.93	
Perceived motivation	Low	50	6.52	255	33.25	462	60.23	<0.01
	High	25	5.13	212	43.53	250	51.33	

Data are presented as frequency and percentage, HBM: Health Belief Model.

**Table 4:** Pap smear and HPV vaccine uptake and suggested reasons behind refusal.

	Variables	N (%)
**What are some reasons people may choose not to take a Pap smear test? #**	It seems embarrassing	712 (53.1)
	It seems painful	635 (47.4)
	Limited or no information in the community	524 (39.1)
	Belief in being healthy	517 (38.6)
	Fear of the screening test results	464 (34.6)
	Fear of the test or a negative experience with a previous examination	364 (27.2)
	Lack of female screener	315 (23.5)
	Low priority	308 (23)
	Lack of provider recommendation	262 (19.6)
	It costs too much	229 (17.1)
	Lack of time	182 (13.6)
	Difficult finding an appointment	150 (11.2)
	Lacking a regular healthcare provider	140 (10.4)
**In your opinion, what are reasons people may choose not to get vaccinated? #**	Don’t trust the vaccine	560 (41.8)
	Fear of infection because of the vaccination	433 (32.3)
	Lack of knowledge or information about the vaccine	352 (26.3)
	Refusal by parents or husband	261 (19.5)
	It is too expensive	143 (10.7)
	Other	94 (7.0)
**In your opinion, what are reasons people may choose to get vaccinated? #**	Influence of family or friends	683 (51)
	Personal decision to receive the HPV vaccine	663 (49.5)
	Recommended by healthcare provider	650 (48.5)
	Public health campaigns and media/educational materials	623 (46.5)
	Personal or family history of HPV or related diseases	623 (46.5)
	Prevention of diseases and infections	610 (45.5)
	Other	44 (3.3)

Data are presented as frequency and percentage, HPV: human papillomavirus, # multiple response question.

**Table 5:** Independent factors affecting the uptake and willingness to receive HPV vaccine.

Willingness to Get Vaccinated Against HPV				Actual Uptake of HPV Vaccine		
Variable	AOR	95% CI	*p*-Value	AOR	95% CI	*p*-Value
**Age**	0.99	0.97–1.00	0.05	0.97	0.94–0.99	0.01
**Health Belief Model Constructs**						
Susceptibility	1.17	1.09–1.26	<0.01	1.21	1.05–1.38	<0.01
Severity	1.03	0.97–1.08	0.34	0.96	0.87–1.06	0.45
Barriers	0.86	0.83–0.90	<0.01	0.82	0.75–0.89	<0.01
Benefits	1.18	1.12–1.24	<0.01	0.97	0.89–1.09	0.54
Motivation	1.03	0.99–1.08	0.14	0.99	0.90–1.07	0.79
**Vaccine Hesitancy**	0.78	0.69–0.90	<0.01	0.48	0.28–0.83	<0.01
**Pap Smear Uptake**	1.78	1.25–2.54	<0.01	1.55	0.74–3.33	0.24

HPV: human papillomavirus, AOR: adjusted odds ratio, CI: confidence interval.

## Data Availability

The raw data supporting the conclusions of this study will be made available by the authors upon reasonable request.

## Abbreviations

CC	Cervical Cancer
HBM	Health Belief Model
HPV	Human Papillomavirus
MOH	Ministry of Health
SFDA	Saudi Food and Drug Authority
SPHA	Saudi Public Health Authority
SPSS	Statistical Package for the Social Sciences
WHO	World Health Organization
